# Hyperthermia-Induced Controlled Local Anesthesia Administration Using Gelatin-Coated Iron–Gold Alloy Nanoparticles

**DOI:** 10.3390/pharmaceutics12111097

**Published:** 2020-11-16

**Authors:** Chien-Kun Ting, Udesh Dhawan, Ching-Li Tseng, Cihun-Siyong Alex Gong, Wai-Ching Liu, Huai-De Tsai, Ren-Jei Chung

**Affiliations:** 1 Division of General Anesthesia, Department of Anesthesiology, Taipei Veterans General Hospital, 201, Sec. 2, Shipai Rd., Taipei 11217, Taiwan; ckting@vghtpe.gov.tw; 2 School of Medicine, National Yang-Ming University, 155, Sec. 2, Linong St., Taipei 11221, Taiwan; 3 Department of Chemical Engineering and Biotechnology, National Taipei University of Technology (Taipei Tech), 1, Sec. 3, Zhongxiao E. Rd., Taipei 10608, Taiwan; dhawan@gate.sinica.edu.tw (U.D.); criswcliu@vtc.edu.hk (W.-C.L.); white9323@gmail.com (H.-D.T.); 4 Institute of Chemistry, Academia Sinica, 128, Sec. 2, Academia Rd., Taipei 11529, Taiwan; 5 Graduate Institute of Biomedical Materials and Tissue Engineering, College of Biomedical Engineering, Taipei Medical University, 250, Wu-Hsing St., Taipei 11031, Taiwan; chingli@tmu.edu.tw; 6 International Ph. D. Program in Biomedical Engineering, College of Biomedical Engineering, Taipei Medical University, 250, Wu-Hsing St., Taipei 11031, Taiwan; 7 Research Center of Biomedical Device, College of Biomedical Engineering, Taipei Medical University, 250, Wu-Hsing St., Taipei 11031, Taiwan; 8 International Ph. D. Program in Cell Therapy and Regenerative Medicine, College of Medicine, Taipei Medical University, 250, Wu-Hsing St., Taipei 11031, Taiwan; 9 Department of Electrical Engineering, School of Electrical and Computer Engineering, College of Engineering, Chang Gung University, 259, Wenhua 1st Rd., Taoyuan 33302, Taiwan; alexgong@mail.cgu.edu.tw; 10 Green Technology Research Center, Portable Energy System Group, College of Engineering, Chang Gung University, 259, Wenhua 1st Rd., Taoyuan 33302, Taiwan; 11 Department of Ophthalmology, Chang Gung Memorial Hospital, Linkou Branch, 5, Fuxing St., Taoyuan 33305, Taiwan; 12 Faculty of Science and Technology, Technological and Higher Education Institute of Hong Kong, 20A, Tsing Yi Road, Tsing Yi Island, New Territories, Hong Kong 999077, China

**Keywords:** lidocaine, hyperthermia, anesthesia, nanoparticles, iron–gold nanoparticles

## Abstract

The lack of optimal methods employing nanoparticles to administer local anesthesia often results in posing severe risks such as non-biocompatibility, in vivo cytotoxicity, and drug overdose to patients. Here, we employed magnetic field-induced hyperthermia to achieve localized anesthesia. We synthesized iron–gold alloy nanoparticles (FeAu Nps), conjugated an anesthetic drug, Lidocaine, and coated the product with gelatin to increase the biocompatibility, resulting in a FeAu@Gelatin–Lidocaine nano-complex formation. The biocompatibility of this drug–nanoparticle conjugate was evaluated in vitro, and its ability to trigger local anesthesia was also evaluated in vivo. Upon exposure to high-frequency induction waves (HFIW), 7.2 ± 2.8 nm sized superparamagnetic nanoparticles generated heat, which dissociated the gelatin coating, thereby triggering Lidocaine release. MTT assay revealed that 82% of cells were viable at 5 mg/mL concentration of Lidocaine, indicating that no significant cytotoxicity was induced. In vivo experiments revealed that unless stimulated with HFIW, Lidocaine was not released from the FeAu@Gelatin–Lidocaine complex. In a proof-of-concept experiment, an intramuscular injection of FeAu@Gelatin–Lidocaine complex was administered to the rat posterior leg, which upon HFIW stimulation triggered an anesthetic effect to the injected muscle. Based on our findings, the FeAu@Gelatin–Lidocaine complex can deliver hyperthermia-induced controlled anesthetic drug release and serve as an ideal candidate for site-specific anesthesia administration.

## 1. Introduction

Nanoparticles have been at the forefront of the pharmaceutical industry for the last two decades, revolutionizing the field of biomedicine [1]. A diversity of metallic nanoparticles such as gold [2,3], silver [4], silicon [5], and polymeric materials [6] have found applications in cancer targeting, as antibacterial agents or in the field of immune-labeling. In addition, metallic oxide nanoparticles such as titanium dioxide and cerium oxide have found applications in drug delivery [7] or cancer therapy [8]. However, the most interesting of applications stem from alloy nanoparticles such as iron–nickel [9], iron–platinum [10], etc. with the property to generate heat upon magnetic stimulation owing to their superparamagnetic nature. Nanotechnological advancements have also enabled the engineering of cancer detection platforms [11,12]. The applications of iron oxide nanoparticles has been widely reported, and numerous studies have found oxide nanoparticles trigger inflammatory response, making them unattractive for in vivo applications [13]. Iron gold alloy nanoparticles have shown tremendous promise for applications in biomedical engineering. The iron component is responsible for the generation of heat upon magnetic stimulation [14,15], while gold increases the biocompatibility of the entire complex [16]. Thus, as shown by previous studies, magnetic hyperthermia can also be employed to precisely control the amount of drug release [17,18]. However, the prospect of using iron–gold alloy nanoparticles for drug release remains unevaluated.

While a plethora of applications of magnetic nanoparticles have been proposed in biology; however, their utilization in the field of anesthesiology is still in its infancy. A recent study by Mantha et al. reported that magnetite nanoparticles can be conjugated to anesthetic drugs such as Ropovacaine to deliver site-specific anesthesia [19]. Furthermore, iron oxide nanoparticles have also been employed as a drug delivery carrier [20]. However, as the nanoparticles are not coated with any biocompatible material, the cytotoxic effects of nanoparticles alone cannot be avoided. We have previously demonstrated that chemodrug release to target cancer cells is achievable using hyperthermia [21]. Further, we used the iron–gold core–shell nanoparticles toward Magnetic Resonance Imaging (MRI) and Optical coherence tomography (OCT) imaging using photostimulation, showing a multitude of nanoparticle properties that can be exploited in biomedicine. In our previous studies that focused on stimulating magnetic nanoparticles to generate hyperthermia, the drug nanoparticle conjugates were uncoated, and thus, a minute cytotoxicity owing to the nanoparticles was observed. An interesting yet unexplored possibility would be coating the drug–nanoparticles conjugate with a biocompatible material. The presence of a biocompatible coating is logically expected to reduce the toxic side effect of nanoparticles [22], increasing the scope of their application for drug release in vivo.

The engineering of nanofilms for anesthetic applications has also been demonstrated [23]. However, practical applications of substrate-based delivery platforms are often not feasible. Recent studies have reported issues such as circulation of the anesthetic drug to unwanted areas of the body, causing side effects [24]. Thus, the issue of limiting the anesthetic drug at the site of action has received critical attention. Surprisingly, no studies have dealt with this issue so far. Strategies such as encapsulating the drug nanoparticle conjugate in a biocompatible material seem more scientifically accurate, where the amount of heat generated precisely controls the amount of drug released. For instance, Wannaphatchaiyong et al. used gelatinized starch films as anesthetic platforms, highlighting the possible use of biocompatible polymeric films for clinical applications [25]. In an ideal scenario, the magnetic nanoparticles can be localized at the intended site of anesthesia by using external magnets, followed by the stimulation via an applied magnetic field (AMF) triggering hyperthermia and leading to the anesthetic drug release in a controlled fashion.

The ability to achieve anesthetic drug release via magnetic field-stimulated hyperthermia in situ is the main hypothesis to this study. Herein, we report the fabrication of FeAu alloy nanoparticles to achieve hyperthermia-mediated anesthetic drug release and explore its applications in the field of therapeutic anesthesia. Briefly, Lidocaine, an anesthetic drug, was conjugated to FeAu nanoparticles. The drug–nanoparticle conjugate was coated with gelatin to improve the overall biocompatibility of drug-conjugated nanoparticles. The cytotoxicity of the FeAu@Gelatin–Lidocaine complex was evaluated in vitro. Furthermore, the ability of FeAu nanoparticles to generate heat in a dose-dependent manner is explored. By using an in vivo rat model, the efficacy of an AMF to stimulate iron–gold alloy nanoparticles (FeAu Nps) to generate hyperthermia, leading to the dissociation of gelatin coating and effective release of anesthetic drug, is demonstrated.

## 2. Materials and Methods

### 2.1. Materials

Ferrous sulfate hepta-hydrate, acetone, and toluene were purchased from Echo Chemicals, Taiwan. Dodecyl dimethyl ammonium bromide, chloroauric acid, 3-mercapto-1-propanesulfonic acid, absolute ethanol, 300 bloom (Type A gelatin), and glutaraldehyde were purchased from Sigma-Aldrich (St. Louis, MO, USA). Xylocaine (2% Lidocaine) was purchased from AstraZeneca. All other chemicals of analytical grade or higher were purchased from either Sigma-Aldrich or Merck (Kenilworth, NJ, USA).

### 2.2. Synthesis and Characterization of Iron Gold Alloy Nanoparticles (FeAu)

The FeAu alloy nanoparticles used in this study were synthesized by the thermal pyrolysis process as previously reported [26]. First, 0.08 mM of dodecyl dimethyl ammonium bromide was dissolved in 10 mL of toluene, poured into a 3-necked flask, and placed on an electric heating plate equipped with a stirrer. The temperature was raised to 110 °C, and the mixture was stirred for 15 min. Then, 0.01 mM ferrous sulfate heptahydrate was dissolved in 0.5 mL of deionized water (DI water), mixed thoroughly, and then slowly injected into the 3-necked flask containing the mixture of dodecyl dimethyl ammonium bromide and toluene. The color of the solution changed from clear to cloudy. After 2 min, 1.5 mL of 0.015 M sodium borohydride was added, and a black suspension was observed in the solution. The mixture was stirred for 20 min. Then, 0.6 mM of 3-mercapto-1-propane sulfate was dissolved in 0.013 M tetra chloroauric acid, and the color of tetra chloroauric acid changed from yellow to clear and then to colorless. Then, 1.5 mL of 0.015 M sodium borohydride was injected into the 3-necked flask, and the color of the solution was observed to change from reddish purple to bright red. The mixture was stirred for 30 min after which 0.5 mL of 0.015 M sodium borohydride was again added and the solution temperature was adjusted to 84 °C, stirred for 3 h, and then allowed to cool down. Then, the solution was centrifuged at 9000 rpm for 10 min. A 4000 Gauss magnet was used to collect the magnetic nanoparticles, dispersed in anhydrous alcohol (ethanol), and added to the centrifuge tube, and the solution was ultrasonicated to uniformly disperse the magnetic nanoparticles. The solution containing nanoparticles was again centrifuged at 9000 rpm for 10 min. This centrifugation step was repeated thrice to remove any trace of bis-dodecyl dimethyl ammonium bromide left behind. The supernatant was discarded, and nanoparticles were dried under vacuum to obtain a dark green powder. The synthesized FeAu were characterized using Transmission Electron Microscopy (TEM, HT-7700, Hitachi, Japan), dynamic light scattering (DLS, ZS90 Plus, Malvern, UK), X-ray Diffractometer (XRD, X’Pert^3^Powder, Malvern Panalytical, UK) and Fourier Transform Infrared Spectroscopy (FTIR, FT720, JASCO, Easton, MD, USA). Magnetic properties of FeAu were analyzed using superconducting quantum interference device (SQUID, MPMS7, Quantum Design, San Diego, CA, USA).

### 2.3. Synthesis and Characterization of Gelatin-Coated Iron–Gold Alloy Nanoparticles Containing Lidocaine Hydrochloride

First, 100 mL of DI water was preheated to 45 °C and 5 g of type A gelatin was added to it and dissolved. The solution was stirred at 45 °C for 30 min. Then, 100 mL of acetone was added to it and stirred. The color of the solution was observed to change from light yellow to white turbid, indicating that gelatin had precipitated. Then, the solution was kept in the refrigerator for 30 min, after which 100 mL of pre-heated water (45 °C) was added to it to dissolve the precipitated gelatin. Three centrifuge tubes were taken and weighed. Then, 1 mL of the gelatin solution was transferred into each of the 3 centrifuge tubes and placed in a 75 °C oven to let it dry for 3 days and then they were weighed again. The difference in weights of the centrifuge tube in the first instance and the second indicates the weight of gelatin/mL. Gelatin solution was diluted to a 2% (*w/w*, pH = 2) solution and heated to 45 °C. One mL of this was added to a 10 mL glass vial and placed in the water bath for 45 min. One mg of iron–gold alloy nanoparticles were dissolved in 1 mL of Lidocaine Hydrochloride (1 M) solution. The mixture was ultra-sonicated to homogeneously disperse FeAu nanoparticles. The glass vial containing gelatin was placed at 45 °C, stirred at 800 rpm, and the solution containing Lidocaine and FeAu was added to it and allowed to mix evenly for 10 min. Then, the heater was turned off, and 3.5 mL of acetone was injected into the solution at the rate of 1.5 mL/minute. The solution was observed to become cloudy. Then, 0.105 mL of glutaraldehyde was added to achieve a concentration of 0.4% in the solution. The solution was further stirred for 3 h. Acetone was extracted using a vacuum pump. The solution was centrifuged at 5000 RCF for 30 min, and the pH was adjusted to 2. The filtrate was taken out, and the amount of Lidocaine present was evaluated by high performance liquid chronography (HPLC). TEM was used to visually observe the gelatin coating over FeAu. DLS was performed to measure the modulation in size and zeta potential in order to confirm the coating of FeAu by gelatin, while FTIR was used to confirm the successful loading of Lidocaine in FeAu and its encapsulation by gelatin.

### 2.4. Characterization of Lidocaine-Containing FeAu Coated with Gelatin

FTIR was used to confirm the conjugation of Lidocaine with gelatin-coated FeAu Nps. Furthermore, to study the gelatin weight loss as a function of temperature, 1 mL of Lidocaine containing gelatin-coated FeAu Nps was placed at −80 °C for overnight. The next day, the conjugated FeAu nanoparticles were vacuum freeze dried. The sample was weighed, and 10 mg of it was taken for Thermogravimetric Analysis (TGA). The samples were heated from 50 to 800 °C, and derivative TGA curves were plotted to express the gelatin weight loss as a function of temperature. All experiments were performed in triplicates. Furthermore, before carrying out HPLC for the estimation of amount of drug released, a variety of concentrations of glutaraldehyde (0.1 to 0.4%) were tested for ideal drug coverage.

### 2.5. Estimation of Amount of Lidocaine Release upon High-Frequency Induction Wave (HFIW) Stimulation

To test the efficacy of FeAu in generating heat upon stimulation with high-frequency induction waves (HFIW), 3 experimental sets were prepared, each containing 0.5 mg, 1 mg, or 2 mg FeAu. The temperature was measured at the beginning of the experiment and an applied magnetic field (AMF; 700–1100 KHz) was turned on. The temperature of all experimental sets was measured every 60 s. To determine the relationship between the FeAu concentration and amount of heat generated, the elevation in temperature was plotted against time. Furthermore, to determine the combined effect of the surrounding temperature and HFIW, 2 experimental sets were prepared. Experimental set 1 was subjected to a water bath, maintained at 37 °C, and then exposed to HFIW, while the experimental set 2 was subjected to an elevated temperature of 40 °C alone. The percentage of FeAu released (as determined through HPLC) was plotted against different experimental conditions.

### 2.6. Cell Culture

L929 (mouse fibroblast, ATCC, USA) cells were cultured in DMEM supplemented with 10% FBS, 100 U/mL penicillin and 100 µg/mL streptomycin. Cell cultures were maintained in an incubator at 37 °C, 95% humidity and 5% CO_2_ atmosphere.

### 2.7. In Vitro Cytotoxicity Analysis Using MTT Assay

To evaluate the cytotoxicity of FeAu@Gelatin–Lidocaine and Lidocaine alone at various concentrations, L929 fibroblasts were seeded in a 96-well plate at a density of 10^4^ cells/mL for 24 h. FeAu@Gelatin–Lidocaine was sterilized under UV light for 30 min in advance and then added to DMEM. The cells were then incubated in different concentrations of either varying concentrations of pure Lidocaine (20, 10, 5 mg/mL) or with FeAu@Gelatin–Lidocaine (10, 5, 2.5 mg/mL). 0.1 g of Teflon was used as a negative control, and 0.1 g latex was used as a positive control. Finally, 1 mL of each experimental set was added to the cells for 24 h and cell viability was assessed using MTT assay.

### 2.8. In Vivo Rat Model for Evaluating Anesthetic Efficiency of FeAu@Gelatin-Lidocaine

Five Sprague-Dawley (SD) rats were randomly used for the in vivo tests (male, weighted 275 ± 25 g). The rats had ad libitum access to standard chow and water at all times. All procedures were performed with prior approval of the Institutional Animal Care and Use Committee of Mackay Memorial Hospital under the number MMH-A-S-106-18. To evaluate the Lidocaine release from the FeAu@Gelatin–Lidocaine conjugate in the absence of HFIW, drug–nanoparticle conjugates were prepared containing a varying amount of Lidocaine (10, 5, and 2.5%) and intravenous injections of drug-conjugated nanoparticles were administered to the tail vein of the rats. The blood was allowed to circulate for 10 min within the body, after which the blood was collected from the heart and the serum was tested for the presence of Lidocaine. Furthermore, to test the efficacy of HFIW in releasing Lidocaine from the FeAu@Gelatin–Lidocaine conjugate and inducing anesthesia, 1 mL of drug–nanoparticle conjugate was administered to the posterior right thigh via an intra-muscular injection. A 4000 Gauss magnet was used to localize the magnetic nanoparticles, and the specific site was subjected to an AMF for 10 min. Then, the treated leg was observed for any visible anesthetic symptoms.

### 2.9. Statistical Analysis

All experiments were performed in triplicates. Datasets different from one another were determined using one-way ANOVA, and the level of significance was set as *p* < 0.05. Data were expressed as mean and standard deviations. Significant experimental values were expressed with a * depicting a *p* value ≤ 0.05, while highly significant experimental values were expressed with ** depicting a *p* value of ≤ 0.01 or *** depicting a *p* value of ≤ 0.001.

## 3. Results and Discussion

### 3.1. Characterization of FeAu Nanoparticles

**Figure 1:**
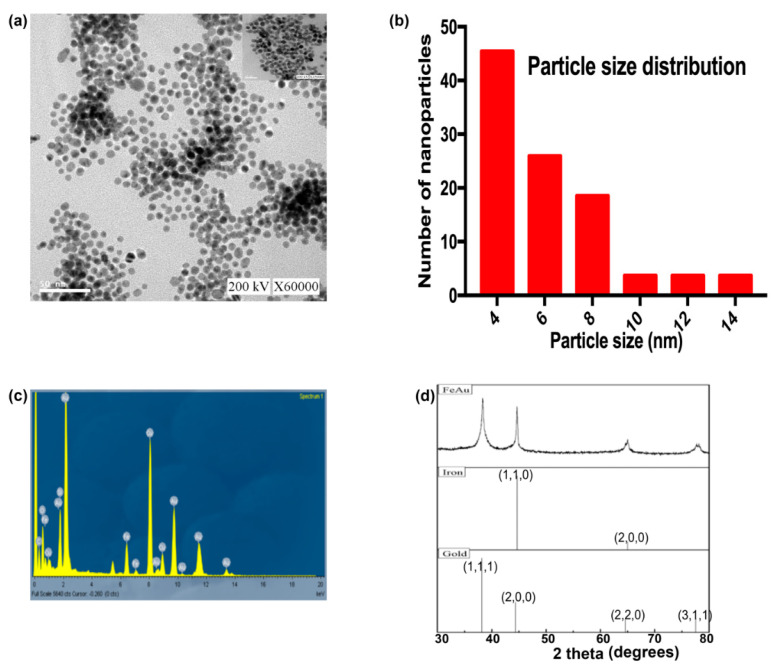
Characterization of FeAu nanoparticles. (**a**) TEM micrographs showing the round morphology of the nanoparticles. (**b**) Graphical representation of size distribution of nanoparticles analyzed using Image J. (**c**) EDS micrograph showing the composition of FeAu nanoparticles. (**d**) XRD analysis of FeAu nanoparticles showing the 2*θ* peaks pertaining to Fe and Au.

In this study, the morphology of FeAu Nps was characterized using TEM, while the composition and size were analyzed through energy-dispersive X-ray spectroscopy (EDS) and dynamic light scattering (DLS), respectively. TEM analysis revealed that FeAu Nps were spherical in shape (Figure 1a), while DLS analysis showed that spherical FeAu Nps had a mean size of 7.2 ± 2.8 nm (Figure 1b). EDS analysis confirmed that the alloy nanoparticles were comprised mainly of iron and gold (Figure 1c). The nanoparticles synthesized in this study were spherical in shape. It will be interesting to observe how the heating ability changes when the nanoparticles are oval or rod-shaped. The effect of nanoparticle shape on its heating power has been the subject of interest for some studies [27]. Another parameter affecting the magnetic properties of nanoparticles is their size. Studies have reported different heating efficiency from differently sized nanoparticles. However, for the sake of simplicity of the experimental designs, we decided to only study the heating efficiency of single-sized nanoparticles in this study. The EDS data showed that the nanoparticles comprised mainly of gold and iron. The presence of Fe and Au in FeAu Nps was further confirmed through XRD, which showed distinct 2*θ* peaks at 38.6, 44.8, and 64.4° that correspond to the (111), (200), and (220) planes of face center cubic (FCC) of gold and the 2*θ* peaks at 43.9 and 64.8 that correspond to the (110) and (200) planes of body-centered cubic (BCC) in iron, thereby further confirming the presence of Fe and Au in FeAu Nps (Figure 1d). These results were firmly consistent with our previous study as well as with those of Krishnamurthy et al. [21,28], thereby confirming the formation of FeAu Nps.

### 3.2. Confirmation of the Formation of FeAu@Gelatin–Lidocaine Drug-Nanoparticle Conjugate

**Figure 2:**
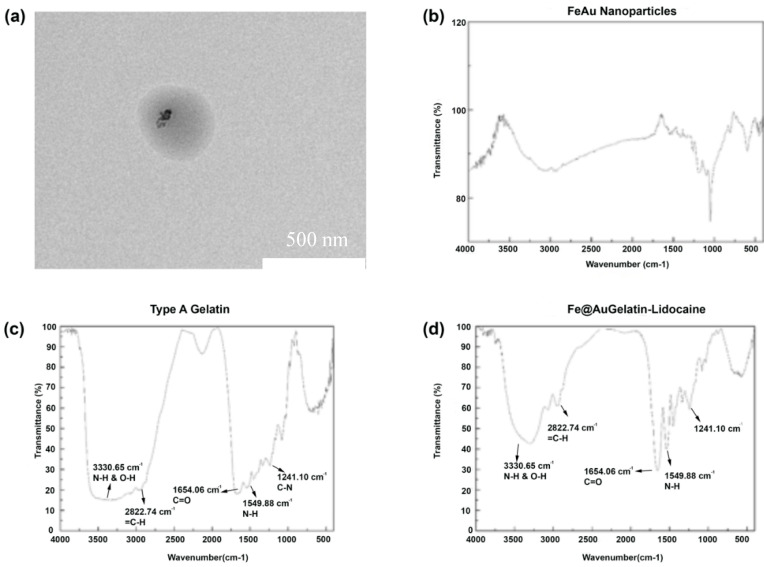
Characterization of gelatin coated FeAu nanoparticles. (**a**) TEM micrograph showing the gelatin coating surrounding FeAu nanoparticles, (**b**) FTIR micrograph of FeAu nanoparticles, (**c**) Type A gelatin and (**d**) FeAu@Gelatin–Lidocaine.

TEM was performed to visually confirm the presence of gelatin around FeAu Nps, which revealed the presence of a dark covering encompassing the FeAu Nps indicating the presence of gelatin over FeAu Nps (Figure 2a). To elucidate this further, DLS was performed to investigate any increment in the size of assumable gelatin-coated nanoparticles. As expected, after incubation of the nanoparticles with gelatin, the average size of the entire complex rose to 348 nm (Supplementary Figure S1). The zeta potential of the complex was maintained at 22 mV (Supplementary Figure S2). The increment in the size confirmed the presence of gelatin around the FeAu nanoparticles. Furthermore, the effect of pH on the size of the FeAu@Gelatin–Lidocaine was also elucidated. At pH 2, the size of the FeAu@Gelatin–Lidocaine complex was measured to be 348 nm; however, when the environmental pH was raised to 7.4, the size of the complex was elevated to 546 nm (Supplementary Figure S3, Table 1). Expectedly, the corresponding zeta potential was observed to be close to 0. This increase can be explained on the basis of the isoelectric point of Type A gelatin, which ranges between pH 7 and 9, thereby leading to the aggregation of gelatin molecules and resulting in the size increase. This observation was also made by Bergo et al. [29]. To further confirm the encapsulation of Lidocaine and FeAu nanoparticles (Figure 2b) in gelatin, Fourier Transform Infrared Spectroscopy (FTIR) was performed. The FTIR of gelatin alone displayed a band at 1241.1 cm^−1^ that can be attributed to weak C–N stretch or the N–H bend of Amide I, while the band at 1549 cm^−1^ can be attributed to the N–H deformation (Figure 2c). The band at 1654 can be attributed to the strong C=O stretching of Amide I, while the band at 2822 cm^−1^ can be due to =C–H, and finally, the band at 3330 cm^−1^ can be attributed to N–H and O–H stretching [30]. The gelatin-coated FeAu–Lidocaine was again subjected to FTIR analysis, which displayed the same bands as gelatin (Figure 2d). The resultant FTIR spectra displayed additional peaks pertaining to Lidocaine [31], indicating the successful conjugation of gelatin with FeAu nanoparticles.

### 3.3. Magnetic Properties of FeAu Nps

**Figure 3:**
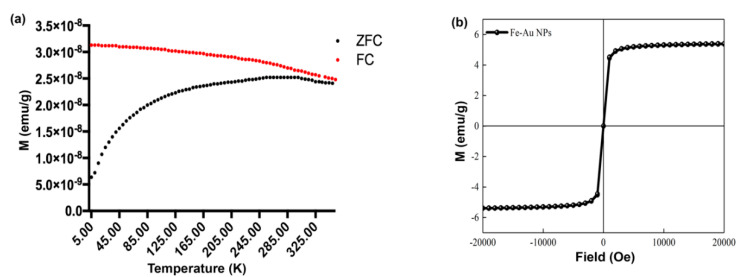
Characterization of magnetic properties of FeAu nanoparticles as analyzed using superconducting quantum interference device (SQUID). (**a**) Zero field cooled (ZFC)/FC curves of FeAu nanoparticles showing the blocking temperature (**b**) M–H curves of FeAu nanoparticles displaying the saturation magnetization.

To investigate the magnetic properties of FeAu Nps, superconducting quantum interference device (SQUID) was used, and the hysteresis loop was analyzed. The induced magnetization was also measured between 5 and 350 K using a fixed magnetic field of 100 Oe. The zero field cooled (ZFC) curve maximized at 300 K (blocking temperature), which indicates the initiation of heat inactivation (Figure 3a). Further, since the blocking temperature is below the room temperature, the nanoparticles are expected to be superparamagnetic in nature. The magnetization was measured between −20,000 G and 20,000 G at 300 K, and the saturation magnetization was found to be 5.5 emu/g (Figure 3b). In addition, no hysteresis was observed. Interestingly, the saturation magnetization observed in our previous study was 3.5 emu/g [26] as compared to 5.5 emu/g in the current study. The observation highlights an interesting aspect that the magnetic properties may be affected by the addition of accessory coatings around nanoparticles.

### 3.4. Thermogravimetric Analysis for the Degradation of Gelatin for Drug Release

**Figure 4:**
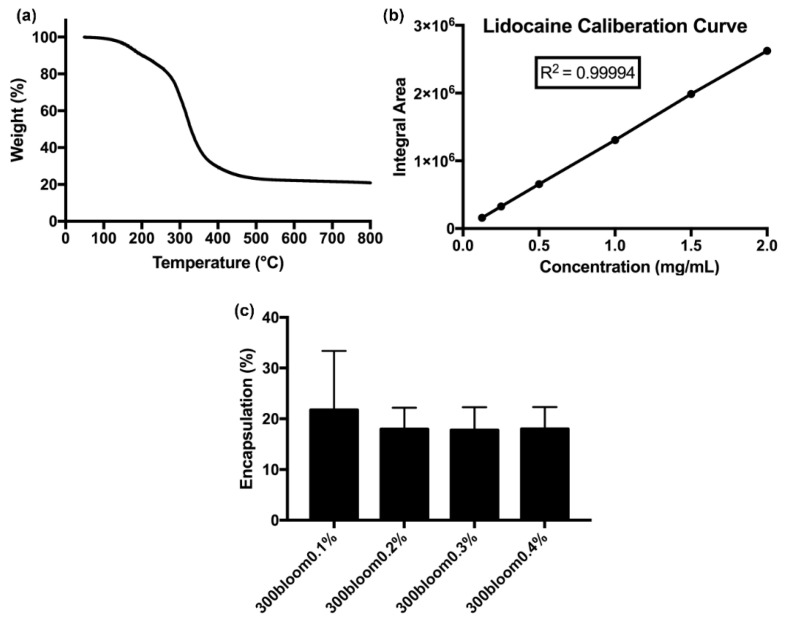
Analysis of gelatin degradation and Lidocaine calibration curve: (**a**) Thermogravimetric analysis (TGA) curve of gelatin degradation, (**b**) Calibration curve of Lidocaine conjugation with FeAu nanoparticles, (**c**) HPLC analysis for estimation of ideal glutaraldehyde concentration for efficient drug–nanoparticle encapsulation.

To evaluate the degradation of gelatin for efficient release of the drug from the FeAu@Gelatin–Lidocaine complex, thermogravimetric analysis (TGA) was performed. The TG thermogram showed that the degradation of gelatin started at a temperature close to 50 °C and continued until the temperature reached 550 °C and then remained constant until 800 °C. Our results are firmly consistent with those of Rahman et al. [32]. Consequently, 97.8% decrease in the mass of the sample was observed when the temperature reached 550 °C, indicating that 95% of the FeAu@Gelatin–Lidocaine complex was composed of gelatin as the outer layer (Figure 4a). Thus, it can be hypothesized that the drug would be effectively released upon degradation of the outer gelatin coating. In order to perform HPLC analysis for the estimation of Lidocaine release in the subsequent experiments, a standard curve was plotted. Then, a 20% Lidocaine Hydrochloride solution was used to prepare the standard solution. A calibration curve with 15, 10, 5, 2.5, and 1.25% Lidocaine was plotted using HPLC (r^2^ = 0.9999, Figure 4b). Then, a FeAu@Gelatin–Lidocaine complex was dissolved in different concentrations (0.1 to 0.4%) of glutaraldehyde (GA) since gelatin coated NPs were cross-linked in these GA concentration, and HPLC was again performed to evaluate the best encapsulation percentage of the FeAu@Gelatin–Lidocaine complex. The encapsulation percentage using 0.1% glutaraldehyde was the highest: close to 22%. Therefore, 0.1% of glutaraldehyde was used in the following HPLC experiments.

### 3.5. Confirmation of Hyperthermia Properties of FeAu Nanoparticles

**Figure 5:**
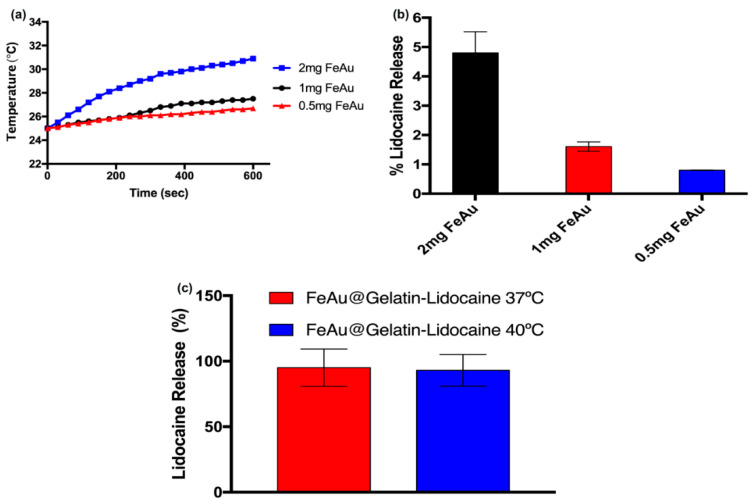
Analysis of hyperthermia properties of FeAu nanoparticles and the corresponding drug release. (**a**) Dose-dependent temperature elevation of the solution containing the FeAu@Gelatin–Lidocaine complex upon stimulation with high-frequency induction waves (HFIW). (**b**) Graphical representation of the percentage of Lidocaine released after a varying amount of FeAu was stimulated by HFIW. (**c**) Graphical representation of percentage of Lidocaine released after incubation of FeAu@Gelatin–Lidocaine in different temperatures prior to HFIW stimulation.

In order to confirm whether FeAu nanoparticles have the ability to generate heat when subjected to a magnetic field, three experimental sets, each containing 0.5, 1, or 2 mg FeAu, were prepared and subjected to HFIW (700–1100 KHz). The samples containing 0.5 or 1mg Fe Au nanoparticles started showing a significant elevation in the temperature only after 100 s in contrast to the samples containing 2 mg FeAu, which triggered a rise in temperature as soon as they were subjected to HFIW (Figure 5a). Furthermore, no significant difference in the temperatures raised by 0.5 or 1 mg was seen until 5 min, indicating that either concentration can be used to achieve similar heating affects. The temperature was maintained at 26.5 and 27.5 °C by 0.5 and 1 mg FeAu nanoparticles after 10 min of HFIW exposure. In contrast, the temperature rose to 31 °C when 2 mg FeAu nanoparticles were exposed to HFIW, indicating the superior heating effect of the nanoparticles. The increment in temperature with the nanoparticle concentration has also been shown by our previous study [26]. The concentration of nanoparticles used in that study was more than 10-fold higher than the one used in the current study; therefore, the corresponding temperature rise (to 40 °C) is justified. Nevertheless, the rise in temperature with nanoparticle amount is ubiquitous. Since different drugs can be conjugated to these nanoparticles in the future, requiring different amounts of energy for their release (in the form of heat), thus, these results indicate that the amount of heat needed for drug release can be well controlled by modulating the amount of conjugated nanoparticles. While the ability of these nanoparticles to generate heat upon magnetic field stimulation was established, the amount of drug released upon exposure to HFIW was still unevaluated. Therefore, in the next step, a varying amount of FeAu@Gelatin–Lidocaine was exposed to HFIW, while Lidocaine release was evaluated using HPLC and expressed as a percentage. Surprisingly, the amount of drug released after exposure to HFIW for 10 min was merely 5%, indicating the subpar drug release (Figure 5b). Some studies in the past have also aimed to achieve the physiological temperature-triggered release of drugs [33,34]. In order to achieve optimal drug release from the drug–nanoparticle conjugate in these studies, the temperature is allowed to rise to 41 °C. However, an increase in temperature beyond 37 °C is expected to trigger cellular apoptosis. Since the intended use of these drug-conjugated nanoparticles is finally in vivo, therefore, 2 mg of FeAu@Gelatin–Lidocaine conjugates were dipped in the water bath maintained at 37 °C or 40 °C, which are close to physiological temperature (37 °C), subjected to HFIW for 5 min, and then taken for HPLC analysis, which revealed that the amount of drug released was close to 98%, indicating that warming of the drug–nanoparticle conjugate prior to HFIW exposure assists in the efficient drug release (Figure 5c). The stimulation of drug release at the physiological temperature also ensures minimal cytotoxic effects to the healthy stroma at the site of anesthesia.

### 3.6. In-Vitro Cytotoxicity Analysis

**Figure 6:**
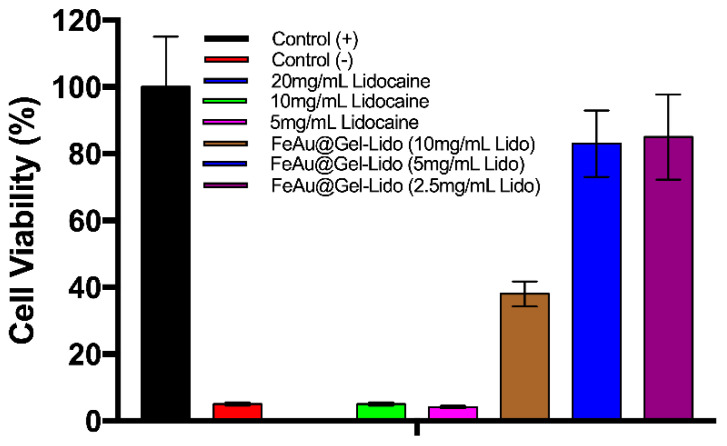
In vitro cytotoxicity assessment of L929 fibroblasts using MTT assay. Graphical representation of cell viability after L929 fibroblasts were incubated in the presence of a varying concentration of Lidocaine or FeAu@Gelatin–Lidocaine.

In the next step, to evaluate the cytotoxicity of the FeAu@Gelatin–Lidocaine complex, L929 fibroblasts were seeded in 96-well plates, and MTT assay was performed, which revealed that all cells died in the experimental groups containing 20 mg/mL pure Lidocaine. Viability was maintained close to 4% in both groups containing a lower dosage of Lidocaine (10 and 5 mg/mL). Similar observations were made by Kuan et al. who showed that after merely 24 h of incubation of cells with 0.75% Lidocaine concentration [35], viability dropped to below 10%. One of the most striking results to emerge in this study was a 9-fold increase in cell viability when the same concentration of Lidocaine (10 mg/mL) was encapsulated in gelatin, indicating the efficiency of gelatin to effectively encapsulate Lidocaine, thereby reducing its cytotoxic effects (Figure 6). A dramatic 20-fold increase was observed when the concentration of Lidocaine encapsulated in gelatin was further decreased (5 mg/mL). These results are conclusive that gelatin successfully shields the cells from coming in direct contact with Lidocaine. A slight decrease in cell viabilities was still observed in experimental groups treated with gelatin-coated drug, which can be attributed to gelatin alone, as the intrinsic properties of gelatin such as charge, molecular weight, and Bloom strength have been seen to modulate its biocompatibility [36]. Nevertheless, it is inarguable that the coating of drug-conjugated nanoparticles with gelatin increases the overall biocompatibility of the complex.

### 3.7. Intravenous Injection and Evaluation of Drug Release in an In Vivo Rat Model

A substantial difference between in vitro and in vivo experiments lies in the presence of diverse biochemicals in the blood, which may account for the degradation of drug–nanoparticle coating. Therefore, in the next step, the FeAu@Gelatin–Lidocaine complex was administered to rats intravenously (tail vein), and the possibility of Lidocaine release was explored. Notably, no HFIW stimulation was provided at this instance. After 10 min of circulation in the body, blood was collected and subjected to HPLC analysis. The retention time of Lidocaine Hydrochloride is close to 6 min (Supplementary Figures S4 and S5). The absence of any convincing peak close to 6 min in the HPLC chromatogram confirmed that no Lidocaine was released from the FeAu@Gelatin–Lidocaine complex. Furthermore, the efficiency of gelatin coating in resisting degradation in a physiological environment, thereby preventing Lidocaine release, was also confirmed.

### 3.8. Intramuscular Injection and Evaluation of Lidocaine Release on HFIW Stimulation In Vivo

Studies have shown in the past that the subcutaneous administration of Lidocaine to the rats reduces response to chemical stimuli, tail flicking, and paw-licking. A multitude of anesthetic effects of Lidocaine are observed, as the drug is not localized to a specific area [37]. The results of our previous experiments demonstrated that the FeAu@Gelatin–Lidocaine complex is stable in the physiological environment in vivo when no HFIW stimulation provided. However, in the current study, we hypothesized that the hyperthermia generated by FeAu Nps upon magnetic field stimulation can be utilized to trigger anesthetic drug (Lidocaine) release through gelatin degradation. Therefore, in the proof-of-concept experiment, we administered an intramuscular injection containing the FeAu@Gelatin–Lidocaine complex to the rat thigh and localized it with a 4000 Gauss magnet. Then, the area of injection was subjected to HFIW stimulation for 10 min, and the rat was unrestrained. As visible in the Supplementary Video S1, the rat can be seen to drag the posterior leg, which was the site of injection. Furthermore, the rat can only be seen to turn left. No right leg movement or turning can be observed, confirming that the injected leg had been anesthetized, restricting the rat from sensing and thereby using it.

## 4. Conclusions

The present study was designed to determine the possibility of using hyperthermia to release Lidocaine from the FeAu@Gelatin–Lidocaine nano-complex and deliver local therapeutic anesthesia effect. We were also interested to elucidate if gelatin coating increases the biocompatibility of the overall system. Our results show that upon exposure to an AMF, the superparamagnetic nanoparticles generated heat in a dose-dependent fashion. In vitro experiments showed that the gelatin coating significantly reduced the cytotoxicity of FeAu@Gelatin–Lidocaine. The drug-conjugated nanoparticles can also be magnetically localized in the targeted area owing to their magnetic properties. The in vivo results showed that upon magnetic stimulation, hyperthermia triggered the Lidocaine release from FeAu@Gelatin–Lidocaine, causing anesthesia at the site of nanoparticle localization. The results also suggested that the amount of Lidocaine release can be controlled by modulating the amount of nanoparticles conjugated to the drug. This study highlights an alternate way to exploit hyperthermia to achieve optimal drug release for therapeutic anesthesia. We anticipate potential applications of this platform in orthopedics and in the fields of biomedical engineering, targeted drug delivery, drug development, and physiology.

## Figures and Tables

**Table 1:** FeAu@Gelatin-Lidocaine size and zeta potential measurement using DLS.

Experimental Group	pH	Zeta Potential (mV)	Size (nm)
FeAu@Gelatin-Lidocaine	2	22 mV	348
FeAu@Gelatin-Lidocaine	7.4	0	546

## Supplementary Materials

The following are available online at https://www.mdpi.com/1999-4923/12/11/1097/s1, Figure S1: Analysis of FeAu@Gelatin size using DLS. The size of gelatin coated nanoparticles increased to 348 nm, indicating successful coating of gelatin; Figure S2: Analysis of FeAu@Gelatin zeta potential using DLS. The results show that the zeta potential of the FeAu@Gelatin complex was 22.0 mV; Figure S3: Analysis of FeAu@Gelatin size using DLS. The results show an increment in size of nanoparticle–gelatin complex in response to the pH variation; Figure S4: HPLC analysis of rat blood before intravenous injection of FeAu@Gelatin–Lidocaine; Figure S5: HPLC analysis of rat blood after intravenous injection of FeAu@Gelatin–Lidocaine in the absence of an external magnetic field. The image shows the absence of peaks pertaining to Lidocaine, confirming that without AMF stimulation, Lidocaine is not released. Video S1: Rat with local anesthesia.
