# Supplementary material for: Hyperthermia-Induced Controlled Local Anesthesia Administration Using Gelatin-Coated Iron–Gold Alloy Nanoparticles
Source: Pharmaceutics. 2020 Nov 16;12(11):1097. doi: 10.3390/pharmaceutics12111097 (PMC7697341; doi:10.3390/pharmaceutics12111097)
Supplement: Supplementary file 1 [file pharmaceutics-12-01097-s001.zip › pharmaceutics-1004554-supplementary/pharmaceutics-1004554-Supplementary.pdf]

pharmaceutics

MDPI

# Supplementary Materials: Hyperthermia-Induced Controlled Local Anesthesia Administration Using Gelatin-Coated Iron-gold Alloy Nanoparticles

**Chien-Kun Ting, Udesh Dhawan, Ching-Li Tseng, Cihun-Siyong Alex Gong, Wai-Ching Liu, Huai-De Tsai and Ren-Jei Chung**

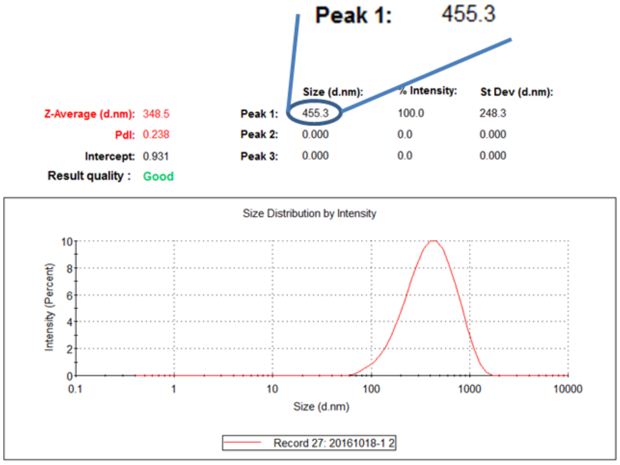


**Figure S1.** Analysis of FeAu@Gelatin size using DLS. The size of gelatin coated nanoparticles increased to 348 nm, indicating successful coating of gelatin.

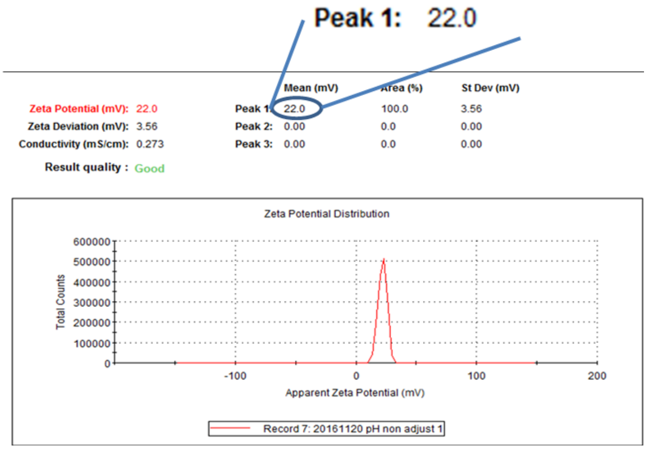


**Figure S2.** Analysis of FeAu@Gelatin zeta potential using DLS. The results show that the zeta potential of FeAu@Gelatin complex was 22.0 mV.

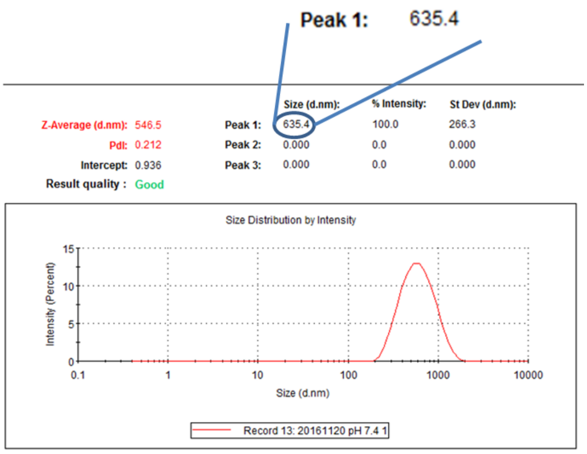


**Figure S3.** Analysis of FeAu@Gelatin size using DLS. The results show an increment in size of nanoparticle-gelatin complex in response to the pH variation.

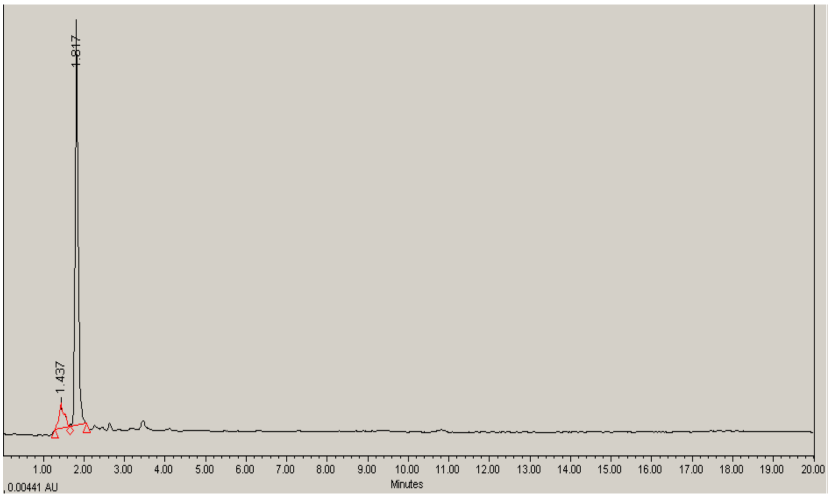


**Figure S4.** HPLC analysis of rat blood before intravenous injection of FeAu@Gelatin-Lidocaine.

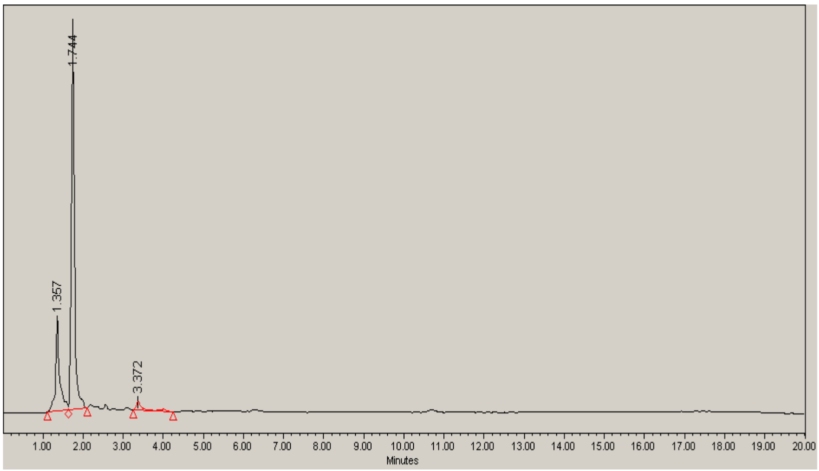


**Figure S5.** HPLC analysis of rat blood after intravenous injection of FeAu@Gelatin-Lidocaine in the absence of an external magnetic field. The image shows the absence of peaks pertaining to Lidocaine, confirming that without AMF stimulation, Lidocaine is not released.
